# The role of simulation modelling in public health policy evaluation

**DOI:** 10.1016/S2468-2667(24)00027-6

**Published:** 2024-03

**Authors:** David D Kim

**Affiliations:** Section of Hospital Medicine, Department of Medicine, University of Chicago, Chicago, IL 60637, USA

As obesity and diet-related chronic diseases become a public health threat in almost all countries, population-based government policies to improve diet quality have been recognised and increasingly implemented as major public health interventions.^1^ When evaluating public health interventions, various methodological approaches—ranging from implementation science to impact evaluation to comparative effectiveness research—could be applied to provide evidence on which interventions work best for whom and under what circumstances.^2^

Evidence acquired from conventional study designs is sometimes insufficient to inform policy decisions. They tend to focus on short-term health outcomes (eg, changes in weight or biomarkers), might not represent a diverse population, and are unlikely to evaluate all policy-relevant options. Simulation modelling can help fill this evidence gap by informing multiple relevant processes, testing a multitude of plausible scenarios that would be impractical and infeasible in trials, quantifying the magnitude of intended and unintended consequences, and having the option to adjust and refine designs before a trial or actual implementation testing in the real world.^3^

In *The Lancet Public Health*, Zoé Colombet and colleagues^4^ used policy simulation modelling to predict the long-term health effects among the adult population in England of implementing the menu calorie labelling policy, which mandates large food businesses to display product energy information for individual items. Conceptually, individual consumers might consume less food or items with lower energy density in response to the calorie information displayed, and the food industry might also reformulate food products sold in large food businesses to contain fewer calories. Existing evidence supports both pathways to some extent, but there is insufficient evidence on how the potential reduction in the overall calorie intake would result in decreased bodyweight and BMI, which in turn reduce the risk of dying from cardiovascular diseases, among many possible outcomes.

The modelling study estimated that the current menu calorie labelling policy would reduce obesity prevalence by 0·31 percentage points (95% uncertainty interval 0·10–0·35; note that 25·9% of adults in England were classified as obese in 2021 to 2022^5^) and prevent 730 cardiovascular disease deaths (430–1300) over the next 20 years when considering the effects on both consumer response and industry reformulation. The estimated policy effect size is relatively small; however, the estimated impact on obesity prevalence is similar to a 2013 modelling study of a 20% tax on sugar-sweetened drinks in the UK, which estimated a step-wise reduction of 1·3% (95% uncertainty interval 0·8–1·7) in the number of adults with obesity^6^ (the 0·31 percentage point reduction in obesity reported by Colombet and colleagues would be equivalent to around a 1·2% reduction, given a prevalence of 25·9%). Considering the small effect size, Colombet and colleagues suggest that expanding the menu calorie labelling policy to all out-of-home food businesses would have much larger effects (2·65 percentage point reduction in obesity prevalence and 9200 cardiovascular disease deaths prevented).

Along with Colombet and colleagues’ important contributions and thoughtful analyses, a few additional points need to be considered to better inform policy decisions. First, the study only measured the health impact regarding reductions in obesity prevalence and cardiovascular disease deaths. The absence of other health outcome measures (eg, diabetes, cardiovascular disease cases, and quality-adjusted life-years) would limit the comprehensive assessment of the comparative advantage of the calorie labelling policy over other public health interventions.

Second, the analysis mainly focuses on projecting health benefits but does not consider economic consequences. It would be imperative to capture economic effects (eg, implementation costs and potential downstream cost-offsets in health-related expenditures) to determine whether additional economic resources are worth the additional health improvement.^7^ Similar US-based studies included formal cost-effectiveness analyses that accounted for the effects on multiple diet-related health and economic outcomes,^8,9^ which is a research gap in the UK.

Finally, the study showed that the effects of the menu calorie labelling policy were similar in the most and least deprived socioeconomic groups, based on the current evidence of equivalent policy effects across socioeconomic groups. As cardiometabolic risk factors are highly correlated and are more prevalent among individuals with low socioeconomic status than in those with high socioeconomic status,^10^ Colombet and colleagues’ other assumption (ie, that the effect of BMI on cardiovascular disease mortality only varies across age groups) means they are likely to underestimate the potential benefits of BMI reduction on cardiovascular disease mortality among the most deprived socioeconomic group. The distributional analyses across socioeconomic groups would have been more precise if the simulation model fully incorporated subgroupspecific effect sizes and surrounding uncertainty.

Despite the known challenges in assessing the long-term effects of public health policy interventions, predictions are often necessary to make an informed, evidence-based decision. When carefully developed and validated policy simulation models are used alongside robust sensitivity and scenario analyses, simulation modelling provides a framework for integrating multiple data sources, reflecting the strength and uncertainty of evidence, incorporating heterogeneous effects across population subgroups, and ultimately identifying cost-effective strategies for improving population health and health equity.
